# *FKBP5* Gene Expression Predicts Antidepressant Treatment Outcome in Depression

**DOI:** 10.3390/ijms20030485

**Published:** 2019-01-23

**Authors:** Marcus Ising, Giuseppina Maccarrone, Tanja Brückl, Sandra Scheuer, Johannes Hennings, Florian Holsboer, Christoph W. Turck, Manfred Uhr, Susanne Lucae

**Affiliations:** 1 Max Planck Institute of Psychiatry, 80804 Munich, Germany; maccarrone@psych.mpg.de (G.M.); brueckl@psych.mpg.de (T.B.); scheuer.sandra@gmail.com (S.S.); florian.holsboer@hmnc.de (F.H.); turck@psych.mpg.de (C.W.T.); uhr@psych.mpg.de (M.U.); lucae@psych.mpg.de (S.L.); 2 kbo-Isar-Amper Clinical Center Munich East, 85540 Munich, Germany; johannes.hennings@kbo.de; 3 HMNC Brain Health GmbH, 80807 Munich, Germany

**Keywords:** depression, antidepressant treatment, HPA axis, gene expression, *FKBP5*, FKBP51

## Abstract

Adverse experiences and chronic stress are well-known risk factors for the development of major depression, and an impaired stress response regulation is frequently observed in acute depression. Impaired glucocorticoid receptor (GR) signalling plays an important role in these alterations, and a restoration of GR signalling appears to be a prerequisite of successful antidepressant treatment. Variants in genes of the stress response regulation contribute to the vulnerability to depression in traumatized subjects. Consistent findings point to an important role of *FKBP5*, the gene expressing FK506-binding protein 51 (FKBP51), which is a strong inhibitor of the GR, and thus, an important regulator of the stress response. We investigated the role of *FKBP5* and FKB51 expression with respect to stress response regulation and antidepressant treatment outcome in depressed patients. This study included 297 inpatients, who participated in the Munich Antidepressant Response Signature (MARS) project and were treated for acute depression. In this open-label study, patients received antidepressant treatment according to the attending doctor’s choice. In addition to the *FKBP5* genotype, changes in blood FKBP51 expression during antidepressant treatment were analyzed using RT-PCR and ZeptoMARK^TM^ reverse phase protein microarray (RPPM). Stress response regulation was evaluated in a subgroup of patients using the combined dexamethasone (dex)/corticotropin releasing hormone (CRH) test. As expected, increased FKBP51 expression was associated with an impaired stress response regulation at baseline and after six weeks was accompanied by an elevated cortisol response to the combined dex/CRH test. Further, we demonstrated an active involvement of FKBP51 in antidepressant treatment outcome. While patients responding to antidepressant treatment had a pronounced reduction of *FKBP5* gene and FKBP51 protein expression, increasing expression levels were observed in nonresponders. This effect was moderated by the genotype of the *FKBP5* single nucleotide polymorphism (SNP) rs1360780, with carriers of the minor allele showing the most pronounced association. Our findings demonstrate that *FKBP5* and, specifically, its expression product FKBP51 are important modulators of antidepressant treatment outcome, pointing to a new, promising target for future antidepressant drug development.

## 1. Introduction

Depression is a very serious and highly prevalent mental disorder. Epidemiological studies suggest an average annual prevalence rate of 5–6% across different cultures, which increases to 10–15% over a lifetime [1]. Depression is also a highly recurrent disorder with more than half of first-episode patients experiencing a second episode or more [2]. It is a highly disabling disorder, ranking third among all causes of time spent living in disability, with only low-back pain and headache disorders causing longer periods of disability [3]. As depression is a multifactorial disorder, genetic and environmental factors also contribute substantially to depression risk and outcome development [4]. Indeed, twin and family studies suggest a 35–40% contribution of genetic factors to disease liability, while the remaining risk variance is best explained by individual environmental events and biographic circumstances [4,5]. Specifically, early adverse life experience has been frequently identified as an important environmental risk factor for adult depression [6,7], and severe and/or long-lasting stressors can trigger new disease episodes in vulnerable individuals [6,8]. 

In fact, acute depression is frequently accompanied by a disturbed stress response regulation, which is indicated, for instance, by elevated endocrine responses to pharmacological challenges of the hypothalamus–pituitary–adrenocortical (HPA) axis, the major stress regulation system [9]. Putatively, the most sensitive challenge test of the HPA axis is the combined dexamethasone (dex)/corticotropin releasing hormone (CRH) test, which evaluates plasma cortisol responses to stimulation with 100 µg CRH under the suppressive effects of 1.5 mg dex [9,10]. This test sensitively detects impaired HPA axis regulation in acute depression, which improves during successful antidepressant treatment. Restored HPA axis regulation after successful treatment as indicated by a normalized cortisol response to the combined dex/CRH test is associated with sustained remission [11,12], while the recurrence of an impaired HPA axis regulation predicts increased relapse risk in remitted patients [13,14]. The glucocorticoid receptor (GR) complex plays a critical role in HPA axis regulation [9,15] as impaired GR signalling results in an attenuated negative-feedback inhibition of the HPA axis, finally leading to chronically elevated glucocorticoid levels. 

The GR function is modulated by chaperone proteins forming a molecular complex that is required for proper ligand binding and receptor activation, as well as transcriptional regulation of the GR target genes [16,17]. The heat-shock protein HSP90 and its cochaperones play a key role in determining the sensitivity of the GR. While HSP90 is essential for GR steroid binding, the cochaperone FK506-binding protein 51 (FKBP51), coded by the *FKBP5* gene, exhibits inhibitory effects by reducing the binding affinity of the GR [18,19]. Genetic variations in the *FKBP5* gene were shown to be associated with the regulation of the HPA axis, with increased depression recurrence and rapid antidepressant treatment response [20,21]. The same genetic variations increased the risk for adult depression [22,23] and for post-traumatic stress disorder [22,24] in individuals reporting early exposure to an adverse environment. These findings suggest the involvement of *FKBP5* gene variants in depression risk and antidepressant treatment outcome; however, the role of *FKBP5* gene expression is yet to be elucidated. Cattaneo and colleagues [25] reported a 11% reduction in leukocyte *FKBP5* RNA expression in patients with major depression (*N* = 74), who responded to eight weeks of antidepressant treatment (citalopram or nortriptyline), while no change was observed in treatment non-responders. These findings were independent of the type of antidepressant used in this study—the selective serotonin reuptake inhibitor (SSRI), citalopram, or the noradrenergic tricyclic antidepressant (TCA), nortriptyline. A recent study did not find changes in leukocyte *FKBP5* RNA expression in female patients with major depression (*N* = 30), who were treated for eight weeks with the SSRI, sertraline, or with the selective serotonin noradrenalin reuptake inhibitor (SNRI), venlafaxine [26]. 

Both studies investigating *FKBP5* gene expression on antidepressant treatment outcome were conducted with relatively small patient samples and did not consider *FKBP5* genotypes previously identified as associated with antidepressant treatment outcome [27]. Therefore, we intended to investigate the effects of change in *FKBP5* gene expression on antidepressant treatment outcome in a large sample of depressed inpatients participating in the Munich Antidepressant Response Signature (MARS) study. In addition, we evaluated the association between *FKBP5* gene expression and impaired HPA axis regulation assessed with the combined dex/CRH test. Finally, we analyzed the moderating effects of rs1360780, a single nucleotide polymorphism (SNP) located in intron 2 of the *FKBP5* gene, for which the most consistent findings on depression risk and antidepressant treatment outcome have been reported [21]. Given the presumed role of restored GR signalling in successful antidepressant treatment in combination with the inhibitory function of FKBP51 on GR sensitivity, we hypothesized (1) that increasing *FKBP5* gene expression is associated with more pronounced dysregulation of the HPA axis and (2) that successful antidepressant treatment is accompanied by a reduced *FKBP5* expression in peripheral blood cells. We further postulated (3) that this effect should be moderated by the rs1360780 genotype previously identified as relevant for antidepressant treatment outcome in depression.

## 2. Results

This analysis included 297 participants of from the Munich Antidepressant Response Signature (MARS) project. MARS is a naturalistic open-label longitudinal treatment study with inpatients suffering from a depressive episode. Patients with a moderate to severe depressive episode were recruited from the hospital of the Max Planck Institute of Psychiatry and collaborating hospitals of Southern Bavaria and Switzerland. Antidepressant treatment outcome was monitored weekly for at least six weeks using the 21-items version of the Hamilton Depression Rating Scale (HAMD-21). Treatment was selected according to the attending doctor’s choice and optimized according to symptom profile, plasma medication levels, and side effects. Mean HAMD-21 depression severity on admission to the hospital (baseline) was 26.1 (SD = 6.0), which decreased to 11.4 (SD = 7.9) after six weeks. Of the total participants, 173 patients (58%) responded to antidepressant treatment as indicated by a reduction of the HAMD-21 score of at least 50%, while the remaining 124 patients were classified as treatment non-responders (ΔHAMD-21 < 50%).

Table 1 presents the demographic and baseline characteristics of treatment responders and nonresponders. No significant group differences were observed (*p* > 0.08). 

Blood samples were collected at baseline and after six weeks. RNA and DNA were extracted from whole-blood samples to evaluate the relative change (percentage from baseline) in *FKBP5* gene expression after six weeks of antidepressant treatment and to analyze the moderating effects of the intronic *FKBP5* variant rs1360780, respectively. In a subgroup of 39 patients (23 responders, 16 non-responders), the relative change in FKBP51 protein levels in blood mononuclear cells was additionally analyzed. FKBP51 protein levels tended to be higher in responders without reaching statistical significance (*p* = 0.080). In a further subgroup of 93 patients (56 responders, 37 non-responders), HPA axis regulation was evaluated at baseline and after six weeks using the combined dex/CRH test. No significant differences in demographic and clinical characteristics between responders and non-responders were found for both subgroups (*p* > 0.175, *p* > 0.272, respectively).

Given the inhibitory effects of FKBP51 on GR sensitivity, we assumed a positive correlation between *FKBP5* RNA expression and cortisol response to the combined dex/CRH test indicating an impaired stress response regulation with increasing *FKBP5* expression. Indeed, we observed significant correlations between *FKBP5* RNA levels and the overall cortisol response to the combined dex/CRH test at baseline (r = 0.214, *p* = 0.044) and after six weeks (r = 0.225, *p* = 0.032); albeit, the size of the effects was small. At the descriptive level, responders to six weeks of antidepressant treatment showed a 52% reduction SD= 145 of the cortisol response to the second combined dex/CRH, while non-responders reached a reduction of only 32% (SD = 115). Given the large variance within the group, this difference did not reach statistical significance (F_1,89_ = 0.53, *p* = 0.468).

**Figure 1:**
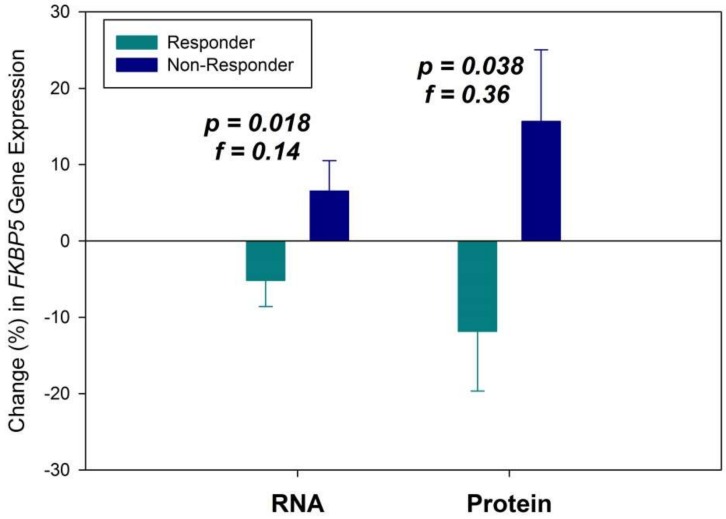
Change (% from baseline) in *FKBP5* gene expression for RNA and protein after six weeks of antidepressant treatment in responders and non-responders. Means ± standard errors of the mean (SEM) are presented.

We observed reduced *FKBP5* gene expression after six weeks in antidepressant treatment responders, while nonresponders presented with increased expression levels (F_1,281_ = 5.71, *p* = 0.018, f = 0.14). In all patients, RNA expression change was significant with a small to medium effect size. The subgroup analysis on change in FKBP51 protein levels revealed the same outcome pattern and was significant (F_1,35_ = 4.64, *p* = 0.038, f = 0.36) approaching the border of a large effect (f = 0.40 [28]) (see Figure 1).

**Figure 2:**
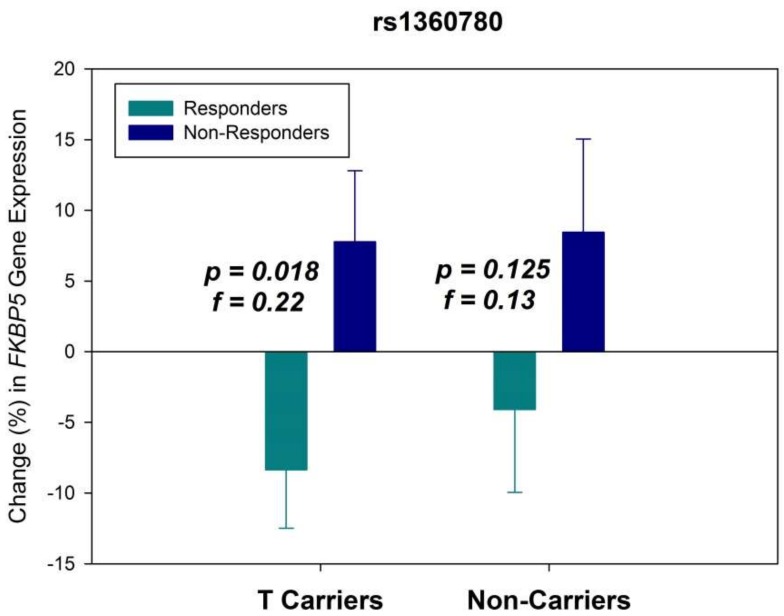
Change (% from baseline) in *FKBP5* gene expression at the RNA level after six weeks of antidepressant treatment in responders and non-responders for patients carrying the minor T allele of rs1360780 and for noncarriers. Means ± SEM are presented.

It was found that while 47.8% of the patients carried the minor T allele of the intronic *FKBP5* variant rs1360780, 52.2% were noncarriers. In agreement with our expectations, patients carrying the T allele tended to show better treatment response with the strongest effect observed after four weeks (odds ratio (OR) = 1.56), which, however, failed to reach statistical significance (*p* = 0.066). This borderline effect was not observed after six weeks (OR = 1.31, *p* = 0.275). To investigate the moderating effect of this genotype, we reanalyzed the response data separately for T allele carriers and noncarriers. The results are presented in Figure 2. Patients of both genotype groups showed the expected effect pattern of reduced *FKBP5* RNA expression in treatment responders. However, the effect was statistically significant only in patients carrying the minor T allele (F_1,122_ = 5.74, *p* = 0.018), presenting with a distinctly larger effect size (f = 0.22) than in the overall analysis (f = 0.14). In noncarriers, the effect pattern was less pronounced (f = 0.13), without reaching statistical significance (F_1,134_ = 2.38, *p* = 0.125). 

## 3. Discussion

While previous findings quite consistently suggested the involvement of *FKBP5* gene variants in depression risk and antidepressant treatment outcome, the role of *FKBP5* gene expression was less clear. Two previous clinical studies [25,26] investigating the association between *FKBP5* RNA expression and antidepressant treatment outcome showed conflicting results. These studies focused on RNA expression and did not analyze FKBP51 protein levels or the effects of *FKBP5* expression on HPA axis regulation. The genotype of the patients was also not considered. In addition, both studies were conducted with small sample sizes (*N* = 74/*N* = 30), and were restricted to specified treatments, which might reduce the generalizability of the findings. To address these limitations, we analyzed *FKBP5* gene expression changes in a large sample of depressed inpatients participating in the MARS study. The open character of the MARS study, which assured optimal treatment for all study participants in combination with liberal inclusion and exclusion criteria, resulted in a high participation rate with only about 15% of invited patients excluded or having refused participation. So it can be assumed that the study sample provided a good representation of patients hospitalized for depression treatment. In addition, we investigated the association between *FKBP5* gene expression and impaired HPA axis regulation in the combined dex/CRH test. Finally, we evaluated the moderating effects of rs1360780, a SNP located in intron 2 of the *FKBP5* gene, for which the most consistent findings on depression risk and antidepressant treatment outcome have been reported [21]. 

First, we could show that *FKBP5* expression correlated with the overall cortisol response to the combined dex/CRH test, although the effect size of the associations was rather small. This is in agreement with previous findings, suggesting that peripheral *FKBP5* RNA expression is induced by elevated glucocorticoid levels in patients with affective and anxiety disorders [20], which could also be confirmed in a human lymphoblastoid cell line model [29]. Contrary to previous findings, we did not find a reduced cortisol response to the combined dex/CRH test associated with response to antidepressant treatment. This could be related to the design of our study. In this study we investigated concomitant changes between HPA axis regulation, *FKBP5* gene expression, and antidepressant treatment outcome, while previous studies on the role of the combined dex/CRH test documented that a normalized HPA axis regulation precedes antidepressant treatment outcome by several weeks [12] and predicts future medium-term disease development [14]. Second, response to six weeks of antidepressant treatment was associated with a reduced *FKBP5* expression. The effect size was small to medium for the change in RNA expression, but bordered on a large effect for the change in protein levels. These findings confirm the results of a previous study reporting reduced peripheral *FKBP5* RNA expression in patients responding to eight weeks of citalopram or nortriptyline treatment [25]. A more recent study did not find changes in *FKBP5* RNA expression in patients treated for eight weeks with sertraline or venlafaxine [26]. However, this study was performed in a very small sample (*N* = 30) and restricted to female patients potentially limiting its power for documenting the general effects of *FKBP5* RNA expression on treatment outcome. In this regard, it is interesting to note that we observed the more substantial effect size for FKBP51 protein levels, which almost bordered on a large effect. This finding supports the biological relevance of the FKBP51 protein as the more proximal marker. Third, after stratifying patients into carriers and noncarriers of the minor allele of rs1360780, the *FKBP5* variant with, thus far, the most consistent findings in depression, the effects were distinctly more pronounced in carriers, while noncarriers showed a similar trend, albeit statistically not significant. It is interesting to note that patients carrying the minor allele also tended to show better treatment response after four weeks of treatment. This borderline effect, however, disappeared after six weeks. It is assumed that the rs1360780 risk variant, despite being intronic, might be associated with an increased glucocorticoid-related induction of *FKBP5* RNA expression [30], presumably, as a result of environmentally triggered changes in DNA demethylation of cytosine-phosphate-guanine (CpG) dinucleotide rich regions in intron 7 of the *FKBP5* gene [31]. Such allele-dependent epigenetic differences in the *FKBP5* gene could have contributed to the observed differences in *FKBP5* expression changes in rs1360780 risk-variant carriers and noncarriers. 

The observed association between reduced *FKBP5* expression and antidepressant treatment response might be indirectly explained by an improved stress response regulation due to diminished inhibitory influences on GR sensitivity leading to a restored HPA axis regulation. However, improved HPA axis regulation evaluated with the combined dex/CRH test was not associated with concomitant antidepressant treatment response in our study suggesting that other, more direct pathways may be involved in the observed association between *FKBP5* gene expression and antidepressant treatment outcome. Indeed, preclinical studies using animal models, cell lines, and human specimens suggested the involvement of several pathways relevant for antidepressant action that are modulated by FKBP51. These pathways include the glycogen synthase kinase-3 (GSK-3) beta pathway [32], autophagy [33], and the modulation of enzymes of the epigenetic machinery [34]. While these findings suggest the potential of a direct causal link between altered FKBP51 and antidepressant action, the exact mechanisms are yet to be elucidated [35]. Nevertheless, FKBP51 inhibitors are currently in development as potential new antidepressant drugs, with early promising findings indicating the anxiolytic and potentially antidepressant effects of such compounds [36,37].

The present study has several strengths, but also some limitations. The strengths of the study are the sample size, which is larger than in previous studies, as well as the additional integration of protein data, genetic data, and a test on HPA axis regulation. There are also several limitations to be mentioned. First, depressed patients with different diagnoses (major depression, recurrent depression, or bipolar depression) were included in this study. However, the distribution of the diagnostic categories did not differ significantly between responders and non-responders (see Table 1). In addition, we repeated all analyses by including the type of diagnosis as additional covariates. The additional diagnostic covariates did not show any significant effects, and all findings could be replicated with similar effect sizes (see Supplementary Table S1). Second, due to the open study characteristic, patients received a variety of antidepressant drugs. Antidepressant treatment was carefully selected considering the current symptom profile of the individual patient, previous treatment history, and the plasma medication levels, with the aim of achieving the best possible antidepressant treatment. While the heterogeneity of the treatment might be a statistical limitation, it is also a strength with respect to the generalizability of the observed findings. In addition, previous studies using the combined dex/CRH test reported homogenous effects of effective antidepressant treatments on HPA axis regulation [11], which presumably can also be expected for FKBP51. Furthermore, we compared the applied classes of antidepressant drug treatment (selective serotonin reuptake inhibitors, tricyclic antidepressants, selective serotonin noradrenalin reuptake inhibitors, noradrenergic and specific serotonergic antidepressants, and other antidepressants) between responders and non-responders at baseline and at six weeks and did not find significant differences (see Supplementary Table S2). Third, *FKBP5* gene expression was analyzed using peripheral blood samples, which might not sufficiently reflect *FKBP5* expression in the pituitary or the brain. While variations in the gene expression pattern between different specimens or tissues cannot be ruled out, there is evidence that glucocorticoid exposure regulates FKBP51 expression via changes in DNA methylation in blood cells and in the brain in a very similar manner [38]. This suggests that peripheral *FKBP5* expression could be a proxy for expression changes in the brain.

## 4. Materials and Methods 

### 4.1. Sample Description and Study Protocol

We recruited 297 patients suffering from a moderate to a severe depressive episode, who participated in the MARS project, for this study. MARS is a naturalistic open-label longitudinal study conducted at the hospital of the Max Planck Institute of Psychiatry and collaborating hospitals in Southern Bavaria and Switzerland to identify predictors for antidepressant drug response and to identify subgroups of depressed patients with common pathology benefitting from personalized treatment [39]. Patients were included during the first week after admission, and antidepressant treatment outcome was evaluated on a weekly basis with the HAMD-21. Only patients with a baseline HAMD-21 score of 14 or higher were included, setting the lower threshold at a moderate depression severity [40,41], while most patients were suffering from severe depression as indicated by an average HAMD-21 baseline score of 26.1 (SD = 6.0). Further exclusion criteria were depressive symptoms secondary to other medical or neurological disorders; presence of manic, hypomanic, or mixed affective symptoms; alcohol dependence or illicit drug abuse; and somatic treatments potentially affecting depression symptoms or HPA axis regulation (e.g., steroid medication). Treatment was selected according to the attending doctor’s choice and optimized according to symptom profile, plasma medication levels, and side effects. Diagnosis according to the World Health Organization International Classification of Diseases, 10th revision (ICD10) [42], was obtained from trained psychiatrists at the end of the hospitalization considering patients reports, reports from relatives, and disease development. 

At study inclusion (baseline) and after six weeks, morning fasting blood samples were collected for DNA extraction (S-Monovette, Sarstedt AG & Co., KG, Nümbrecht, Germany) and RNA extraction (PAXgene tubes; QUIAGEN GmbH, Hilden, Germany). In a subgroup of patients (*N* = 39), additional morning fasting serum samples (S-Monovette, Sarstedt AG & Co., KG, Nümbrecht, Germany) were collected at both time points—baseline and after six weeks—with mononuclear cells immediately isolated (ACCUSPIN System Histopaque-1077; Sigma-Aldrich, Merck KGaA, Darmstadt, Germany) for protein analysis. In another subgroup of 93 patients, a combined dex/CRH test was performed at baseline and after six weeks (the day after blood sample collection). All patients provided oral and written consent prior to study inclusion after all study details were explained. Ethical approval was provided by the permanent ethics committee of the Medical Faculty at the Ludwig–Maximilian University Munich, Germany (approval code: 318/00, 21/03/2001).

### 4.2. Laboratory Analysis

DNA was extracted using the Gentra PureGene extraction kit (QUIAGEN GmbH, Hilden, Germany) and genotyping was performed as part of a series of larger genotyping projects using Illumina Beadchip technology (Illumina Inc., San Diego, CA, USA). Rs1360780 was selected as the representative variant for the *FKBP5* risk genotype (minor allele frequency: 0.28; test for deviation from Hardy–Weinberg equilibrium: *p* = 0.667). RNA was isolated and purified with RNeasy kits (QUIAGEN GmbH, Hilden, Germany); expression analysis was performed with real-time polymerase chain reaction (RT-PCR) method using a TaqMan gene expression assay (Applied Biosystems Deutschland GmbH, Darmstadt, Germany). *FKBP5* RNA quantification was calculated against the activity of four housekeeping genes (*beta-glucuronidase*, *hypoxanthine–guanine phosphoribosyltransferase 1*, *phospholipase A2*, and *TATA-box binding protein*) using the delta cycle threshold (CT) method [43] for each housekeeping gene. Resulting deltas were then averaged across the four housekeeping genes showing excellent concordance for both time points (average intraclass correlation: *r* = 0.937). A ZeptoMARK^TM^ reverse phase protein microarray (RPPM) platform (Zeptosens AG, Witteswil, Switzerland) was used for FKBP51 protein profiling following the manufacturer’s standard protocol [44] with array readout and quantification performed using the analysis software ZeptoVIEW 3.0 (Zeptosen AG).

The combined dex/CRH test was conducted as previously described [14]. Briefly, 1.5 mg dex was administered orally at 11 p.m. the evening before CRH stimulation. Blood samples were drawn the next day at 3:00, 3:30, 3:45, 4:00, and 4:15 p.m. while the subjects remained supine throughout the test. Within 30 s, after the collection of the first sample, 100 µg human CRH was injected. The cortisol response to the dex/CRH test was assessed by the total area under the curve (AUC) using the trapezoid rule across plasma cortisol concentrations of all sampling points. Plasma cortisol was determined by radioimmunoassay (ICN Biomedicals, Carson, CA, USA; detection limit 0.3 ng/mL).

Plasma medication levels were analyzed using liquid chromatography followed by mass spectrometry at the clinical laboratory of the Max Planck Institute of Psychiatry. Depending on the type of medication, metabolites of the active compound were also assessed to obtain a complete picture of the relevant drug concentrations. This information was available to the attending doctor to assist in finding the optimal drug dosage for the individual patient.

### 4.3. Statistical Analyses

Patients were classified as treatment responders and non-responders depending on the observed percent change in the HAMD-21 total score between baseline and six weeks with score improvements of 50% or more defined as response. Changes in *FKBP5* RNA expression and FKBP51 protein levels were also calculated as percent changes from baseline. RNA and protein values deviated from a normal distribution (Kolmogorov–Smirnov goodness-of-fit test, *p* < 0.01). To reduce skewness, scores were log-transformed to base 10 for the statistical analysis. Differences between responders and non-responders were evaluated by means of chi-square (categorical data) and *t* tests (continuous data) for demographic and clinical variables. Change in HAMD-21 scores over time, as well as baseline and change scores for the cortisol response to the dex/CRH test for *FKBP5* RNA expression and for FKBP51 protein levels were evaluated with analyses of covariance, controlling for the effects of sex and age as potential confounding variables. Cohen f scores [28] were calculated as effect-size measures. Associations between *FKBP5* RNA expression and the cortisol response to the combined dex/CRH test were expressed with partial correlation coefficients, which were controlled for the effects of sex and age. The level of statistical significance was set to *p* = 0.05. Means, standard deviations, or standard errors of uncorrected/ untransformed values are reported in the tables and figures. All analyses were performed with PASW Statistics 18 (IBM, Armonk, NY, USA).

## 5. Conclusions

We were able to demonstrate that successful antidepressant treatment outcome in depressed patients is accompanied by a reduction in *FKBP5* gene and FKBP51protein expression, particularly in those patients, who are carrying the risk allele of the *FKBP5* variant rs1360780. These findings further suggest an important role for *FKBP5* and FKBP51 in antidepressant treatment outcome and point to a new, promising target for future antidepressant drug development. However, further studies are warranted to fully understand the mechanism behind the observed effects.

## Figures and Tables

**Table 1:** Demographic and baseline characteristics of responders and non-responders to six weeks of antidepressant treatment.

Characteristics	Responders *N* = 173	Nonresponders *N* = 124	*p*
Female sex (%)	77 (44.5%)	63 (50.8%)	0.284
Mean age (SD)	48.8 (14.0)	47.0 (13.4)	0.270
Diagnosis ^1^ (%)			0.121
F31	22 (12.7%)	7 (5.6%)	
F32	36 (20.8%)	30 (24.2 %)	
F33	115 (66.5%)	87 (70.2%)	
HAMD-21 ^2^ (SD)	26.5 (6.63)	25.5 (5.07)	0.156
FKBP5 RNA ^2^ (SD)	1.83 (0.88)	1.75 (0.65)	0.404
FKBP51 ^2,3^ (SD)	0.035 (0.07)	0.031 (0.06)	0.080

^1^ Diagnosis according to ICD10, ^2^ at baseline, ^3^ available only in a subgroup of *N* = 39 patients: 23 responders, 16 non-responders.

## Supplementary Materials

Supplementary materials can be found at http://www.mdpi.com/1422-0067/20/3/485/s1.

## Abbreviations

AUC	Area under the curve
CRH	Corticotropin releasing hormone
CpG	Cytosine-phosphate-guanine
Dex	Dexamethasone
DNA	Deoxyribonucleic acid
GR	Glucocorticoid receptor
*FKBP5*	FK506-binding protein 5 gene
FKBP51	FK506-binding protein 51
HAMD-21	Hamilton Depression Rating Scale, 21-items version
HPA	Hypothalamus–pituitary–adrenocortical
HSP90	Heat-shock protein 90
ICD10	International Classification of Diseases, 10th revision
MARS	Munich Antidepressant Response Signature
OR	Odds ratio
RNA	Ribonucleic acid
RPPM	Reverse phase protein microarray
RT-PCR	Real-time polymerase-chain-reaction
SD	Standard deviation
SEM	Standard error of the mean
SNP	Single nucleotide polymorphism
SNRI	Selective serotonin noradrenalin reuptake inhibitor
SSRI	Selective serotonin reuptake inhibitor
TCA	Tricyclic antidepressant
