# Supplementary material for: FKBP5 Gene Expression Predicts Antidepressant Treatment Outcome in Depression
Source: Int J Mol Sci. 2019 Jan 23;20(3):485. doi: 10.3390/ijms20030485 (PMC6387218; doi:10.3390/ijms20030485)
Supplement: Supplementary file 1 [file ijms-20-00485-s001.pdf]

Supplementary Material

Supplementary Table 1. Analyses of covariance (ANCOVA) on change in *FKBP5* RNA and FKBP51 protein expression after six weeks of treatment in responders and non-responders, with the type of diagnosis (Major Depression, Recurrent Depression, Bipolar Depression) included as additional covariates.

ANCOVA[1]	F_{df}	p value	*Effect size f*
**Change in FKBP5 RNA expression**	$\mathbf{F_{1,279} = 5.62}$	**0.018**	**0.14**
**Change in FKBP51 protein expression**	$\mathbf{F_{1,34} = 4.25}$	**0.047**	**0.35**
**Change in FKBP5 RNA expression in T allele carriers**	$\mathbf{F_{1,120} = 4.98}$	**0.028**	**0.20**
Change in FKBP5 RNA expression in T allele non-carriers	$F_{1,132} = 2.06$	0.153	0.12

[1] Significant effects printed in bold-face.

Supplementary Table 2. Configuration Frequency Analysis (CFA) to compare the multivariate pattern of prescribed antidepressant medication classes (selective serotonin reuptake inhibitors, tricyclic antidepressants, selective serotonin noradrenalin reuptake inhibitors, noradrenergic and specific serotonergic antidepressants, other antidepressants) between responders and non-responders at baseline and after six weeks of treatment.

CFA	X^2_{df}	p value
Medication at baseline	$X^2_{df=23} = 24.59$	0.372
Medication after 6 weeks	$X^2_{df=20} = 24.19$	0.234
